# Response of *Hypothenemus hampei* Ferrari (Coleoptera: Curculionidae: Scolytinae) parasitized by the nematode *Metaparasitylenchus hypothenemi* Poinar (Tylenchida: Allantonematidae) to different colors of light

**DOI:** 10.2478/jofnem-2024-0011

**Published:** 2024-04-08

**Authors:** M. Simota-Ruiz, A. Castillo-Vera

**Affiliations:** El Colegio de la Frontera Sur, Carretera Antiguo Aeropuerto Km. 2.5, Tapachula, CP 30700, Chiapas, México

**Keywords:** attraction, behavior change, host-parasite relationship, insect vision, interaction, parasitism

## Abstract

*Metaparasitylenchus hypothenemi* is a nematode that naturally parasitizes *Hypothenemus hampei* in a coffee-producing region in Chiapas, Mexico. This study investigated changes in the attraction of parasitized borers to light. We compared the attraction of adult *H. hampei* females (parasitized and uninfected) to 14 different light wavelengths (350–670 nm) with a control (570 nm, yellow) under laboratory conditions. The response ranges of non-parasitized and parasitized borers were 370–650 nm and 340–650 nm, respectively. The attraction curve showed a similar shape in both borer groups (parasitized and non-parasitized), but a wide wavelength range (380–590 nm) attracted more parasitized than non-parasitized borers. The maximum response of the uninfected borers occurred at 520 nm (green), while parasitized borers exhibited three response peaks (380 nm, violet; 460 nm, blue; 520 nm, green). Parasitized borers were significantly more attracted to green light (520 nm) than to the control. The altered attraction to light in borers parasitized by *M. hypothenemi* is discussed from the perspective of possible host manipulation and the natural prevalence of this parasite.

## Introduction

The nematode *Metaparasitylenchus hypothenemi* Poinar (Nematoda: Allantonematidae) was first reported to parasitize adults of *Hypothenemus hampei* Ferrari (Coleoptera: Curculionidae: Scolytinae) in a commercial coffee plantation in southeastern Mexico (Castillo et al., 2002). Since its discovery, this parasitic nematode has been detected in various coffee plantations throughout Mexico (Pérez et al., 2015). Reproductive females of *M. hypothenemi* and/or several hundred juveniles, and eggs can be recognized in the hemocoel of parasitized adult females (Poinar et al., 2004). *Metaparasitylenchus hypothenemi* is an obligate parasite that exploits its host to complete a part of its life cycle (Poinar et al., 2004), and infection induces sterility and reduces host longevity (Castillo et al., 2019; Castillo et al., 2002). Obligate parasitic nematodes rarely kill their host, although they can modify their physiology and behavior (Grucmanová and Holuša, 2013). Parasitic nematodes can affect various parameters in scolytids, such as adult emergence (Ashraf and Berryman, 1970; Hoffard and Coster, 1976), flight activity (Kinn and Stephen, 1981), locomotive activity (Schutgens et al., 2013), fecundity (MacGuidwin et al., 1980), longevity (Schutgens et al, 2013), survival (Grucmanová and Holuša, 2013) and gallery construction (MacGuidwin et al., 1980). Adult confused flour beetles (*Tribolium confusum* Jacquelin du Val; Coleoptera: Tenebrionidae) infected with *Protospirura muricola* (Gedoelst, 1916) (Nematoda: Spiruridae) stayed in illuminated areas (Schutgens et al., 2013). Although the attraction of *H. hampei* to light has received relatively little attention, it was demonstrated that females can discriminate between different wavelengths in the visible light spectrum (Chong et al., 2006). The coffee berry borer completes the largest part of its life cycle inside coffee berries, but females use visual cues to spread through coffee plantations and localize new berries (Giordanengo et al., 1992; Mathieu et al., 1997). We examined the attraction of parasitized and uninfected coffee berry borers to the visible light spectrum in the context of the “behavioral manipulation hypothesis” (Poulin, 1995).

## Materials and Methods

Approximately 1,000 residual fruits infested with *H. hampei* were collected in a commercial coffee plantation (*Coffea canephora* Pierre ex. A. Froehner; Rubiaceae) located in the municipality of Cacahoatán, Chiapas, Mexico (N 15°00′27.6″, W 92°09′51.2″; 564 masl). *H. hampei* females used in the bioassays were obtained from residual fruits placed in plastic containers (3.8 l) with ventilated lids. A sample of 100 borers was used to determine the natural parasitism rate at the collection site, which was 50%. A bioassay was designed using a glass T-shaped olfactometer (20 mm internal diameter; Facultad de química, UNAM, México) to assess light color attraction by *H. hampei* females as previously described (Brown et al., 1998; Castillo and Rojas, 2020). The evaluated wavelengths were 340, 350, 370, 380, 400, 420, 460, 490, 520, 540, 590, 640, 650, and 670 nm (treatments), which were compared to a control light source (570 nm, yellow) for which most insects have photoreceptors (Briscoe and Chittka, 2001). The intensity of both light sources (halogen lamps of 150 W, Osram, Fiber-Lite PL-750) was adjusted to 2.3 mV using a solar cell and measured with a digital multimeter (Master® Brand, Mod. MAS830L). The bioassays were carried out between 4:00 and 8:00 PM in an experimental room with the lights switched off, a temperature of 27 ± 3°C, and a relative humidity of 70 ± 8%. In each bioassay, a borer was placed in the central area of the T-tube and simultaneously illuminated by two different light sources with different wavelengths (treatment vs. control). After starting each bioassay, the borers were allowed to move freely inside the chamber for 3 min. At the end of the test, the room lights were switched on, and the location of borers near both filters (control and treatment) was recorded (Castillo and Rojas, 2020). The location of the treatment and control was reversed after every bioassay to eliminate directional bias. A female borer was considered attracted when walking more than 5 cm from the initial point towards any of the light sources. Five insects per treatment were observed daily in a random order on ten different days, and each insect was considered as a replicate. A total of 50 females were observed for each wavelength evaluated, summarizing 700 borers to the 14 wavelengths evaluated, and each one was tested only once. After the bioassays, borers were dissected to determine the parasitism by *M. hypothenemi* as described by Pérez et al. (2015). The identification of nematodes was based on the morphological characteristics of the parasitic juvenile and adult stages (Poinar et al., 2004). The number of borers attracted to each treatment was compared with the control using Pearson's Chi square test (α = 0.05) with Williams’ correction (Sokal and Rohlf, 1995). All analyses were performed with the R statistical software package version 3.6.2 (R Core Team 2019).

## Results and Discussion

**Figure 1:**
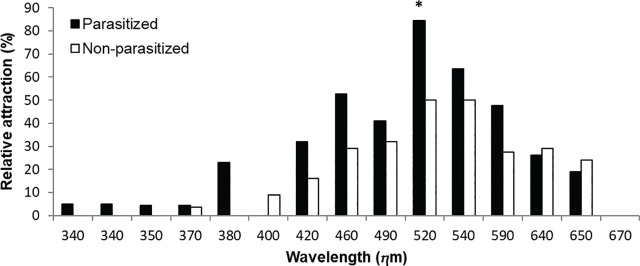
Relative attraction of CBB (parasitized with *Metaparasitylenchus hypothenemi* and non-parasitized) to 14 light wavelengths compared to the control (570 nm). The asterisk-labeled treatment was statistically different to the control using the χ^2^ test (*P* = 0.01). Relative attraction (%) was calculated using the number of borers that chose the treatment and control, applying the formula: [(treatment) (100) / (treatment + control)].

Beetles used in bioassays were parasitized at a rate of 48.4% (339/700; Table 1). No beetles remained in the central area of the T-tube during the bioassays. The shape of the light spectrum response curve was similar in parasitized and uninfected borers, and both were attracted to visible light. In the range 380–590 nm (Fig. 1; Table 1), a significantly higher number of parasitized borers (n = 78) were attracted to light than uninfected borers (n = 53) (χ^2^ =14.2; df =1; *P* = 0.01). Parasitized borers (n = 99) responded to light at 340–650 nm (except for 400 nm), while the response range for non-parasitized borers (n = 73) was 370–650 nm (except for 380 nm; Fig. 1; Table 1). A single attraction peak, defined as a point that stands out above the rest, was exhibited at 520 nm (green) by nematode-free borers, while three attraction peaks at 380 nm (violet), 460 nm (blue), and 520 nm (green) were displayed by parasitized borers (Fig. 1; Table 1). Significantly more parasitized females (n=27; χ^2^ = 6.8, df = 1, *P* < 0.01) were attracted to 520 nm (green) than to the control (n = 5; 570 nm, yellow). We did not observe a relationship between parasitism and attraction to the control (Fig. 1; Table 1) in the other evaluated wavelengths. This work reports for the first time that the nematode *M. hypothenemi* induces a change in the attraction of *H. hampei* to light. The shape of the uninfected *H. hampei* response to the light curve was like what was reported in a previous work (Chong et al., 2004). However, our results show that the sensitivity of this pest to light increases when it is parasitized by *M. hypothenemi*, particularly to green. Color perception by insects has been previously discussed by other authors (Briscoe and Chittka, 2001), and the cues used by *H. hampei* to locate coffee berries have been a matter of debate. Several authors suggest that the coffee berry borer is attracted by red (Damon, 2000). However, others have commented that the role of coffee berry color by itself remains to be elucidated (Vega et al., 2009). However, the pest's color response observed in the present work has also been reported for other bark beetle species during host plant location (Atkins, 1966). Other beetle groups, such as fireflies (Cronin et al., 2000) and scolytins, are also attracted by color. For example, *Dendroctunus pseudotsugae* Hopkins and *Ips paraconfusus* Lanier have photoreceptors for blue, green, and UV light (Groberman and Borden, 1981; Briscoe and Chittka, 2001). Also, the bark beetles *Dendroctonus pseudotsugae* Hopkins and *Trypodendron lineatum* Olivier were attracted to green light in laboratory tests (Groberman and Borden, 1981). However, our results show that the shape of the response curve and maximum sensitivity levels for this pest are different from those exhibited by other scolytins. Some authors have pointed out that *H. hampei* uses visual stimuli to locate its host plant (Giordanengo et al., 1992; Mathieu et al., 1997), but so far, the cues used by this pest to locate coffee berries remain unelucidated (Vega et al., 2009). This specialist herbivore requires specific cues to locate its host plant, as successful localization of a host plant is crucial for herbivorous insects (Silva and Clarke, 2020). This pest builds galleries into the endosperm of the coffee berries and spends a large part of its life cycle in darkness. *Hypothenemus hampei* females are stimulated by the rain to emerge from the residual berries, while males die inside the berry (Mathieu et al., 1997). The females fly without apparent direction during the hours of maximum solar radiation to search for new coffee berries (Baker et al., 1992; Giordanengo, 1992). Light reflected by foliage could provide important visual stimuli for locating coffee plants, in which green and yellow are the predominant colors (> 450nm; Menzel, 1979). The change in sensitivity to light reported here could increase locomotor activity in parasitized borers, stimulating the early abandonment of coffee berries by females, the dispersal of the parasite, and the infection of new hosts. Detailed studies are required to determine the causes that induce this behavior in parasitized borers. The impact of *M. hypothenemi* on *H. hampei* has been relatively underexplored, although it is known that this parasite induces sterility in adult females by reducing the number of oocytes (Castillo et al., 2019). Previous studies have analyzed the influence of light color on the behavior of *H. hampei* parasitoids (Castillo and Rojas, 2020), although no reports about changes in light sensitivity were available for this pest until now. Other authors have commented that some parasites that induce behavioral changes in their hosts can cause adaptive manipulation of the host (Poulin, 1995). It is possible that the behavioral changes observed in *H. hampei* confer an advantage to *M. hypotenemi*, since it is known that some insect parasites can alter the light perception of their hosts to their own advantage (Obayashi et al., 2021).

**Table 1:** Response of coffee berry borer females (parasitized by *Metaparasitylenchus hypothenemi* and uninfected) attracted by 14 different wavelengths (nm) compared to the control (570 nm) inside a T-shaped olfactometer. The frequency of response (n=50) to each treatment and control was compared using a square Chi test.

**Wavelength**	**Parasitized**		**Non-parasitized**		**χ^2^**	**P**
	
	**Treatment**	**Control**	**Treatment**	**Control**		
340	2	34	0	14	13.89	1
350	1	21	0	28	30.64	0.44
370	1	22	1	26	32.03	0.45
380	8	27	0	15	1.24	0.08
400	0	16	3	31	0.19	0.54
420	8	17	4	21	1.75	0.18
460	10	9	9	22	2.78	0.09
490	9	13	9	19	0.41	0.52
520	27	5	9	9	6.75	0.01
540	14	8	14	14	0.92	0.33
590	10	11	8	21	2.12	0.14
640	5	14	9	22	0.04	0.83
650	4	17	7	22	0.18	0.66
670	0	26	0	24		1

*Metaparasitylenchus hypotenemi* is an obligate parasite that was found parasitizing coffee berry borer adults in residual coffee berries approximately 20 years ago (Poinar et al., 2004). This discovery, however, triggered some questions: When does the infection occur? How does the parasite enter the berries? Because it is important for obligate parasitic nematodes to live close to their host, the inside of coffee berries represents an appropriate habitat and offers the possibility of finding new hosts for this parasite. In this sense, light color not only seems important for the host plant's localization by the pest but could also play an important role in the dispersal of the parasitic nematode and in the localization of new hosts. Changes in light sensitivity could lead to increased flight activity in parasitized borers. They transport the nematode to other berries, reducing the time necessary to locate a new host. This change in behavior, in combination with the cryptic habits and the inter-harvest cycle of the pest, could explain how this parasitism has prevailed for more than twenty years in this region.
